# A Spontaneous Mutation in *tfo*X Impairs Bioluminescence in *Photobacterium kishitanii* ANT‐2200

**DOI:** 10.1111/mmi.70104

**Published:** 2026-07-25

**Authors:** Capucine Le Cam Ligier, Gwenola Simon, Charlotte Berthelier, Corinne Valette, Elisa Lefeuvre, Marc Garel, Laurie Casalot

**Affiliations:** ^1^ Aix Marseille Université Université de Toulon, CNRS, IRD, MIO Marseille France; ^2^ Sorbonne Université CNRS, Station Biologique de Roscoff Roscoff France

**Keywords:** bacterial bioluminescence, gene regulation, *lux‐rib* operon, *tfoX*

## Abstract

Bioluminescence in luminous Vibrionaceae is often regulated by quorum sensing, yet several *Photobacterium* strains emit light independently of cell density, suggesting alternative regulatory inputs. Here, we characterized DimLum, a spontaneous, stable low‐luminescence mutant of *Photobacterium kishitanii* ANT‐2200. RNA‐seq showed a pronounced downregulation of the *lux–rib* operon, while whole‐genome sequencing identified a 20‐nucleotide deletion in *tfo*X predicted to truncate the C‐terminal region of TfoX. Complementation with the wild‐type *tfo*X gene restored *lux–rib* expression and bioluminescence, linking this mutation to the DimLum phenotype. The mutant also displayed a limited transcriptional response, including upregulation of a small set of genes such as a Cro/C1‐type regulator. Together, these results identify TfoX as an important component of the regulatory network supporting bioluminescence in *P. kishitanii* .

## Graphical abstract

A frameshift mutation in *tfo*X generates a truncated regulator associated with reduced *lux–rib* expression and low luminescence. Complementation with wild‐type *tfo*X restores transcription and light production, supporting a role for TfoX in regulating bioluminescence independently of quorum sensing.

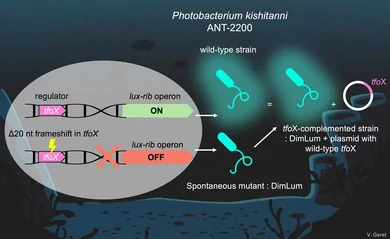

## 1 Introduction

Bioluminescence is widespread among marine bacteria and plays well‐established ecological functions in symbioses with fishes and cephalopods, including counterillumination camouflage, predator deterrence and prey attraction (Haddock et al. 2010; Widder 2010). In free‐living bacteria, however, its ecological significance remains less clear (Ramesh and Mohanraju 2017; Tanet et al. 2020). Most luminous marine bacteria belong to the Vibrionaceae, particularly the genera *Vibrio*, *Aliivibrio*, and *Photobacterium* (Ast et al. 2007; Fuertes‐Perez et al. 2021; Moi et al. 2017; Urbanczyk et al. 2011). Light production depends on the *lux* operon: *lux*AB encodes luciferase, *lux*CDE encodes enzymes required for synthesis of the fatty aldehyde substrate, and *lux*G contributes to flavin reduction (Brodl et al. 2018; Meighen 1993; Sung and Lee 2004). In *Photobacterium*, *lux* operons are particularly diverse and may include accessory genes such as *lux*F or a downstream riboflavin biosynthesis module, *rib*EBHA, which may contribute to variation in operon architecture, light output and metabolic integration (Brodl et al. 2020; Gould et al. 2023; Machado and Gram 2017; Urbanczyk et al. 2008).

In well‐studied luminous Vibrionaceae, such as *Aliivibrio fischeri* and *Vibrio campbellii* , bioluminescence is regulated by quorum sensing (QS), which coordinates *lux* expression with cell density (Baldwin et al. 1989; Bellissimo et al. 2024; Dunlap 2014; Qin et al. 2007; Stevens and Greenberg 1997; Verma and Miyashiro 2013). These systems can include local LuxI/LuxR regulators as well as broader Vibrionaceae pathways involving signal receptors, LuxU/LuxO, Qrr small RNAs, and downstream master regulators (Ball et al. 2017). Therefore, the absence of *lux*I/*lux*R genes near the *lux* operon does not necessarily exclude QS regulation. However, several *Photobacterium* species display luminescence patterns inconsistent with classical cell‐density‐dependent control, suggesting that other mechanisms may influence *lux–rib* expression (Dunn et al. 2015; Katznelson and Ulitzur 1977; Tanet et al. 2019). *Photobacterium kishitanii* ANT‐2200, isolated from Mediterranean waters at 2200 m depth, produces strong bioluminescence. During a fermenter experiment, we isolated a spontaneous, stable low‐luminescence mutant, DimLum. This mutant provided a genetic entry point to investigate the determinants associated with reduced light production in this strain. Unlike typical dark mutants in vibrios, which often arise from disruptive lesions in the *lux* operon itself (O'Grady and Wimpee 2008), DimLum showed no structural alteration in the light production genes after initial verification of this locus. To our knowledge, this makes DimLum the first low‐luminescent variant in *Photobacterium* described without disruption of the light production genes. We combined transcriptomic, whole‐genome sequencing and genetic complementation to identify the genetic determinant responsible for this regulatory alteration. This approach identified *tfo*X, encoding a TfoX/Sxy‐family regulator associated with natural competence in Vibrionaceae (Metzger et al. 2019; Wu et al. 2015), as a genetic determinant of full bioluminescence in *P. kishitanii* .

## 2 Results and Discussion

### 2.1 A Spontaneous Reduction of Luminescence Reveals a Global Shutdown of the lux–rib Operon

The spontaneous low‐luminescence mutant DimLum displayed growth kinetics comparable to those of the wild‐type strain (WT). Both strains reached similar maximal optical densities (ODmax = 1.99 ± 0.16 for DimLum and 1.63 ± 0.02 for WT) and showed indistinguishable specific growth rates (Qx = 0.0109 and 0.0106 min^−1^, respectively; Wilcoxon test, *p* > 0.05; Table S1). These results indicate that DimLum did not show impaired growth under the tested conditions. In contrast, light production was strongly reduced in DimLum compared with WT, both for maximal raw bioluminescence (BLmax = 2.4 × 10^3^ ± 1.9 × 10^3^ vs. 1.04 × 10^5^ ± 3.0 × 10^3^ photons s^−1^) and for OD‐normalized light production (qBLmax = 7.6 ± 8.4 vs. 617.1 ± 53.3 photons s^−1^ ODU^−1^). The very low and highly variable qBLmax value observed in DimLum therefore indicates a strong reduction in luminescence but does not support consistent light detection throughout growth.

Across early, mid‐ and late exponential phase, transcriptomic analysis revealed a marked and coordinated downregulation of the *lux–rib* operon in DimLum (Figure 1). The *lux* and *rib* genes (Figure 1A) were the only strongly downregulated loci genome‐wide, with fold changes (FC) ranging from approximately 4‐ to 40‐fold (log_2_FC from −1.97 to −5.37; adjusted *p* value < 0.05; Table S2; Figure 1B). No mutation was detected in the *lux–rib* structural genes or their regulatory region, indicating that the reduced luminescence in DimLum is associated with transcriptional downregulation rather than structural disruption of the light production locus.

**FIGURE 1:**
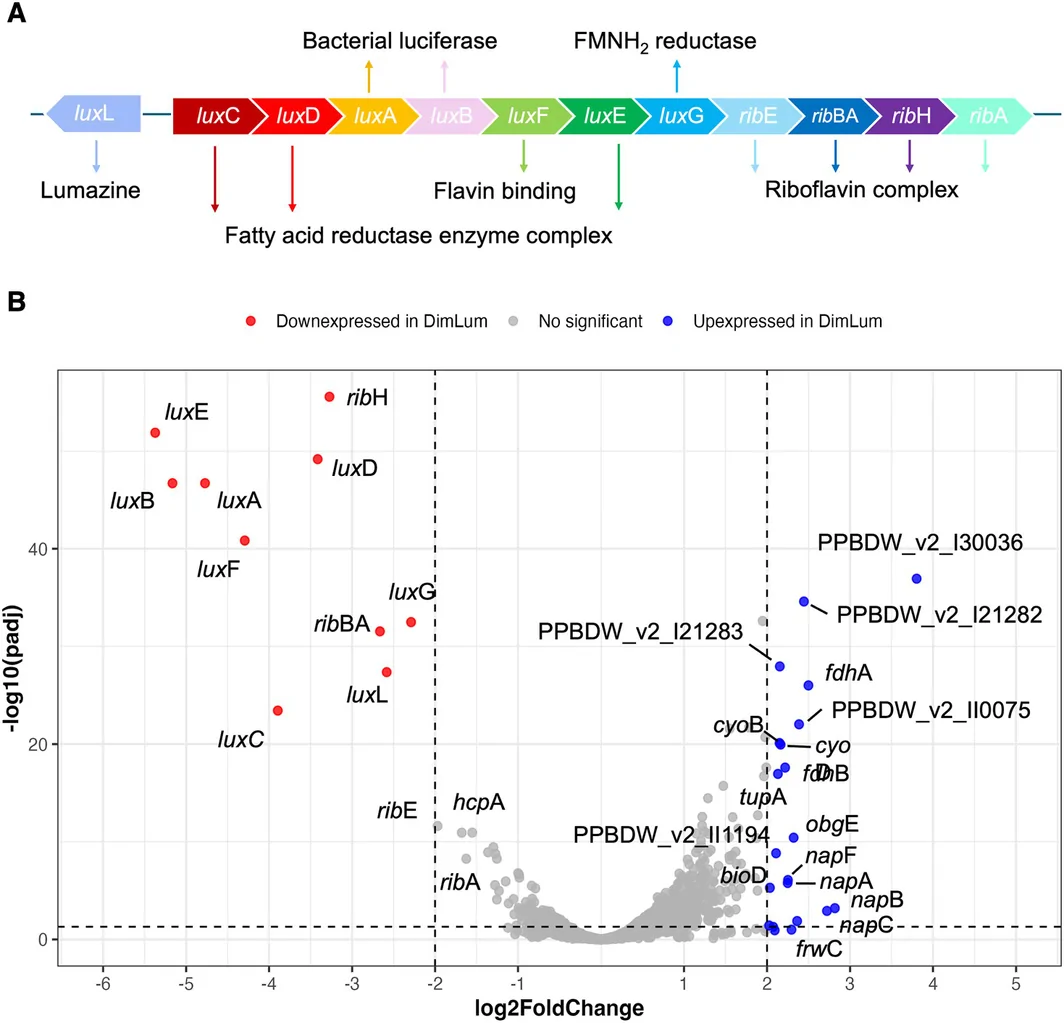
(A) Organization of the light emission‐involved genes in *Photobacterium kishitanii* ANT‐2200. (B) RNA‐seq differential expression analysis comparing WT and DimLum, displayed as a volcano plot. Each point represents a gene, plotted according to log_2_FC and –log_10_(adjusted *p* value). The analysis was performed by combining data from three time points (T1–T3) (Table S7) corresponding to different stages of the growth curve, which showed highly similar differential expression patterns with no substantial variation in the set of differentially expressed genes. Genes not significantly differentially expressed are shown in gray, genes significantly upregulated in DimLum are shown in blue, and genes significantly downregulated in DimLum are shown in red. Vertical dashed lines indicate log_2_FC thresholds, and the horizontal dashed line indicates the significance cutoff (adjusted *p* value < 0.05).

### 2.2 A Single Frameshift in tfoX Underlies a Reduced Luminescence

Whole‐genome sequencing of WT and DimLum identified 19 high‐confidence variants differentiating the two genomes, including synonymous, upstream, and missense variants, as well as two variants predicted to have high‐impact (Table S3). One high‐impact variant affected an annotated tRNA‐Lys gene (Bloom‐Ackermann et al. 2014), whereas the other corresponded to a 20‐nucleotide deletion in PPBDW_v2_I20952, annotated as a *tfo*X homologue (Figure 2). Given the coordinated downregulation of the *lux–rib* operon and the known regulatory role of TfoX/Sxy proteins in Vibrionaceae (Metzger et al. 2019; Wu et al. 2015; Yamamoto et al. 2011), the *tfo*X mutation was considered the most plausible candidate underlying the DimLum phenotype.

**FIGURE 2:**
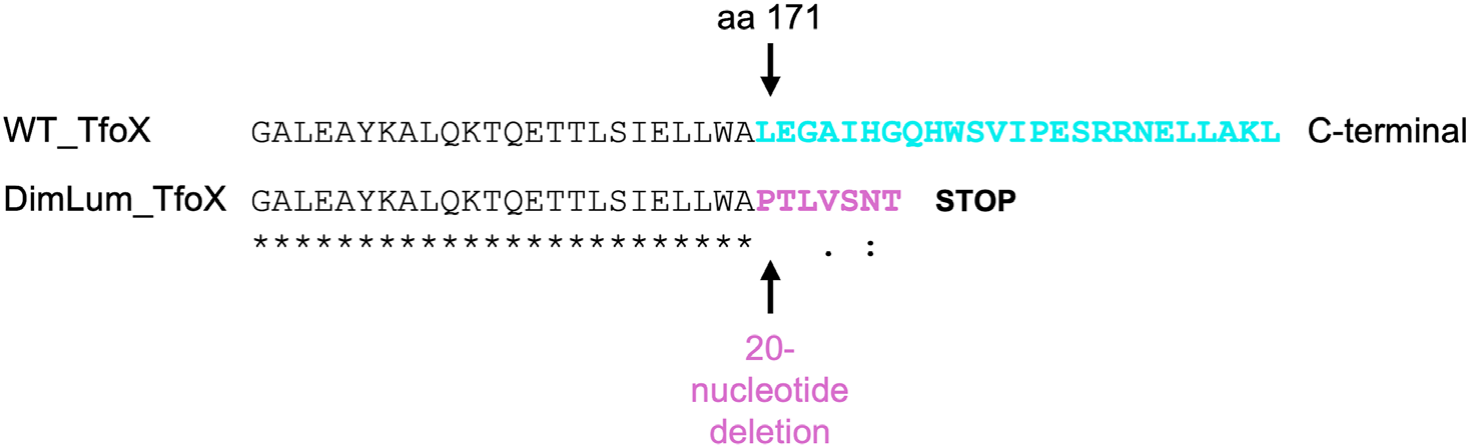
Sequence alignment of the C‐terminal region of TfoX from the wild‐type strain (WT_TfoX) and the DimLum mutant (DimLum_TfoX), generated using Clustal 2.1. A 20‐nucleotide deletion causes a frameshift at amino acid position 171, resulting in an altered amino acid sequence followed by a premature stop codon.

TfoX/Sxy proteins have been characterized as broad regulators in several *Gammaproteobacteria*, including Vibrionaceae (Metzger et al. 2016). They contribute to coordinated transcriptional programs, including competence‐related pathways, and can act with cAMP–CRP to activate genes containing CRP‐S‐type promoter elements (Cameron and Redfield 2006; Lo Scrudato and Blokesch 2013; Søndberg et al. 2019; Sun et al. 2013). In *Vibrio cholerae* , *tfo*X expression is connected to chitin‐dependent competence induction and is posttranscriptionally enhanced by the small RNA TfoR (Dalia et al. 2014; Søndberg et al. 2019; Sun et al. 2013; Yamamoto et al. 2011). The *P. kishitanii* ANT‐2200 protein encoded by PPBDW_v2_I20952 fits this family: the 195‐amino acid WT sequence was most similar to TfoX/Sxy proteins from *Photobacterium*, including a *Photobacterium phosphoreum* homologue showing 90.77% identity over the full query sequence, and more distant full‐length *Vibrio* homologues showing approximately 60%–62% identity. *P. kishitanii* ANT‐2200 also encoded a second TfoX‐like protein, but this paralogue was more divergent, sharing 32.8% identity and 52.4% positives with the DimLum‐associated TfoX/Sxy protein over a 189‐aa aligned region. These comparisons support the annotation of the mutated gene as a TfoX/Sxy homologue and indicate that this strain encodes an additional, more divergent TfoX‐like paralogue.

Despite the broad regulatory roles described for TfoX/Sxy proteins in other Vibrionaceae (Metzger et al. 2019), the transcriptional signature associated with the DimLum gene was highly focused under the tested conditions: 99.25% of the transcriptome remained unchanged, whereas the *lux–rib* operon was strongly downregulated (Table S2). Competence‐associated genes, such as *pil*A were only weakly affected or unaffected (Table S4), suggesting that the mutation does not simply abolish all TfoX‐related functions under these conditions. This focused response may reflect the growth conditions used, partial activity of the altered TfoX protein, or a contribution of the second TfoX‐like paralogue. Together, these results link the DimLum *tfo*X frameshift gene to reduced bioluminescence in *P. kishitanii* ANT‐2200, while leaving open whether TfoX affects the *lux–rib* operon directly or through intermediate regulators.

### 2.3 Wild‐Type tfoX Restores Bioluminescence and Selected Transcriptional Changes

Complementation of DimLum with the WT *tfo*X gene restored bioluminescence to near‐WT levels (Figure 3A). Growth of the complemented strain, DimLum(pCLCL‐*tfo*X1), remained broadly comparable to WT and DimLum, although its specific growth rate was slightly reduced (Qx = 0.0081 min^−1^ vs. 0.0106 and 0.0109 min^−1^ for WT and DimLum; Table S1), suggesting a mild cost associated with *tfo*X expression from the complementation plasmid. In contrast, bioluminescence was restored to near‐WT levels (Figure 3A). Maximal light output reached 8.4 × 10^4^ ± 5.1 × 10^3^ photons s^−1^, approaching WT levels, while the maximal specific rate of light production (qBLmax) was not only restored but exceeded that of the WT strain (902.8 ± 133.0 vs. 617.1 ± 53.3 photons s^−1^ ODU^−1^). Because OD_600_ is an indirect proxy for biomass, the apparent over‐restoration of normalized light production was interpreted cautiously.

**FIGURE 3:**
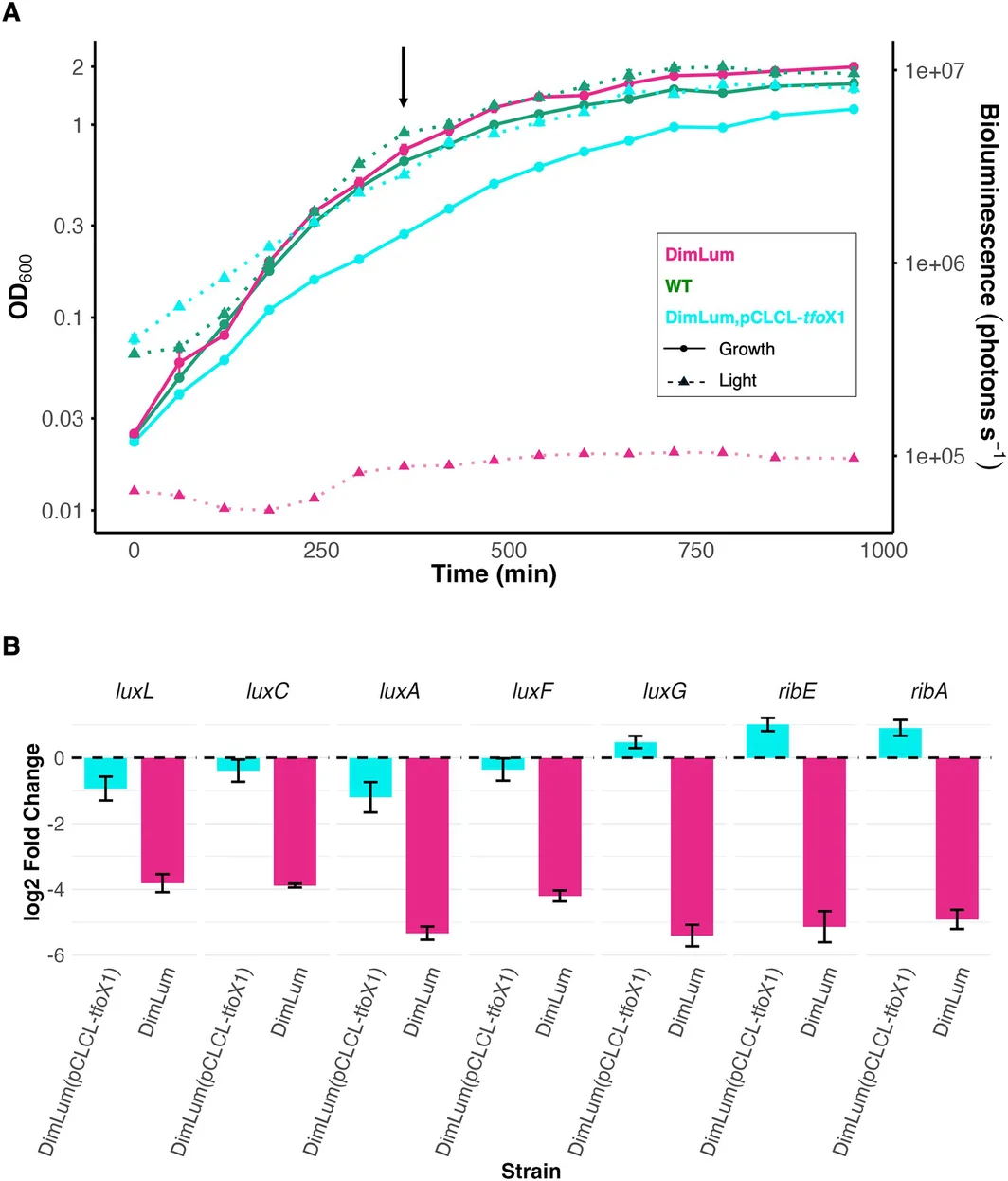
(A) Growth (solid lines, left *y*‐axis) and bioluminescence (dashed lines, right *y*‐axis) kinetics of the wild‐type strain WT (green), the low‐luminescent mutant DimLum (magenta), and the *tfoX*‐complemented strain, DimLum (pCLCL‐*tfo*X1) (blue). Growth was monitored by OD_600_, and bioluminescence was measured as photon emission. For visualization purposes, the bioluminescence signal of the DimLum strain was rescaled near the lower limit of the graph. The black arrow indicates the time point used for RNA sampling (T2 = 360 min), corresponding to the samples subsequently analyzed by RT‐qPCR. (B) RT‐qPCR analysis of selected genes from the *lux–rib* operon. Expression levels are shown as log_2_FC relative to the WT reference strain (set to 0), after normalization with the housekeeping gene *rpoD*. Bars represent mean values across biological replicates, with error bars indicating the standard error of the mean (SEM). The *tfoX*‐complemented strain is shown in blue and the DimLum strain in pink.

Consistent with phenotypic recovery, RT‐qPCR showed restoration of *lux* and *rib* gene expression in the *tfo*X‐complemented strain (Figure 3B; Table S4). A similar trend was observed throughout growth (Figure S1). The empty‐vector control did not restore luminescence or *lux–rib* expression, indicating that recovery depended on WT *tfo*X. Together, these data show that the WT *tfo*X gene is sufficient to restore *lux–rib* transcription and bioluminescence in the DimLum background.

Amongst the genes upregulated in DimLum, the strongest signal corresponded to PPBDW_v2_I30036, encoding a putative Cro/C1‐type helix‐turn‐helix regulator (log_2_FC ≈ 3.35) (Table S2). Cro/C1‐family regulators are often associated with transcriptional switches (Konovalova et al. 2014; Mahmoud and Chien 2018; Schubert et al. 2007), and related regulators have been implicated in phenotypic switching and luminescence variation in *Photorhabdus luminescens* (Eckstein et al. 2021). This Cro/C1‐like regulator may therefore represent an intermediate component linking the altered *tfo*X gene to the low‐luminescence transcriptional state.

RT‐qPCR analysis of selected non‐lux genes supported the RNA‐seq results (Table S2) and showed that the expression of three genes was reproducibly shifted toward WT levels upon tfoX complementation: PPBDW_v2_I30036, encoding the Cro/C1‐like regulator; PPBDW_v2_II0075, encoding a predicted metal‐dependent protease; and PPBDW_v2_II1194, encoding a protein of unknown function (Figure 4). Other genes showing intermediate changes, including respiratory or redox‐associated genes such as *fdh*A, *cyo*B, and *nap*A, may reflect downstream physiological consequences of the low‐luminescence state rather than direct TfoX‐dependent regulation. This interpretation is consistent with observations in *Photobacterium leiognathi* KNH6, where variation in bioluminescence was associated not only with the *lux*, *lum*, and *rib* operons, but also with genes linked to metabolism (*suc*A, *mdh*) and transcriptional regulation (*ssp*A) (Dunn et al. 2015).

**FIGURE 4:**
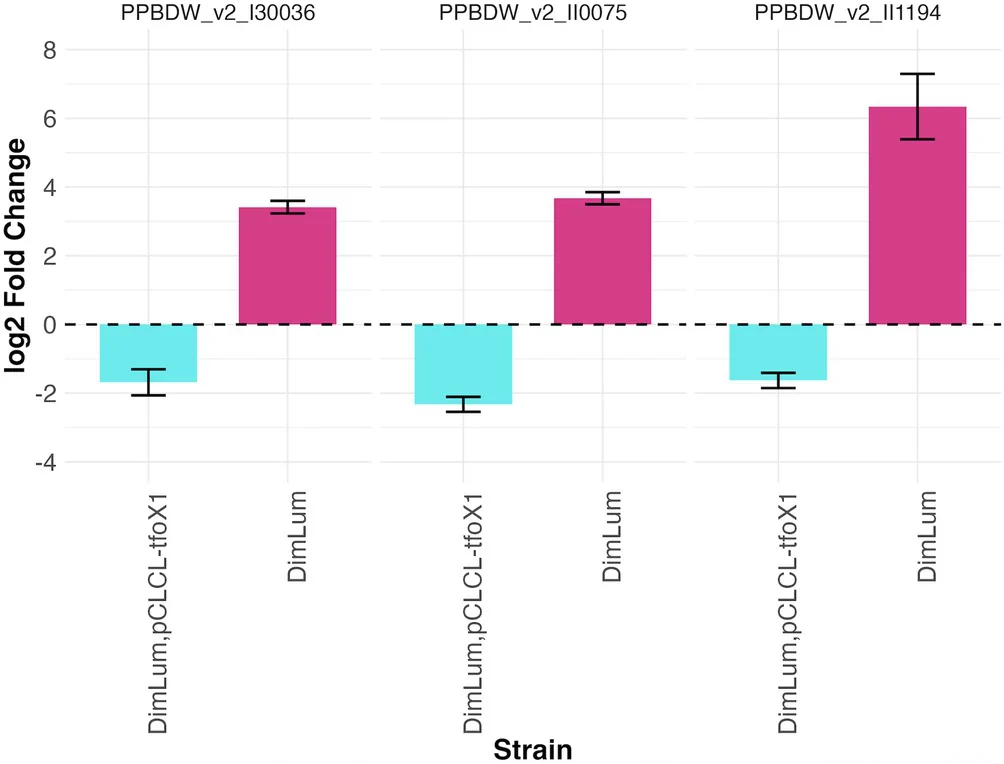
*tfo*X complementation restores the transcriptional profile of selected target genes across growth time points. Log_2_ FC in gene expression are shown for three representative genes (PPBDW_v2_I30036, PPBDW_v2_II0075, and PPBDW_v2_II1194) in the DimLum mutant and the complemented strain, DimLum (pCLCL‐*tfo*X1), relative to the wild‐type (WT) background at the T2 timepoint, after normalization with the housekeeping gene *rpo*D. Bars represent mean log_2_ FC values, and horizontal error bars indicate variability (±SEM). The vertical dashed line at log_2_FC = 0 denotes no differential expression compared to WT. The *tfo*X‐complemented strain is shown in blue and the DimLum strain in pink.

Overall, these results support the involvement of *tfo*X in the DimLum phenotype and suggest a regulatory link with the *lux–rib* bioluminescence operon. Under the tested conditions, the transcriptional response remained limited, with *lux–rib* expression as the main affected output. This study provides evidence that spontaneous variation in *tfo*X is associated with altered bioluminescence in *P. kishitanii* ANT‐2200. Whether this effect is direct or mediated by intermediate regulators, such as the Cro/C1‐like factor identified here, remains to be determined. Future work should test TfoX binding, promoter activity in targeted *tfo*X mutants, and the contribution of candidate regulators to this pathway.

## 3 Experimental Procedures

### 3.1 Bacterial Strains, Culture Conditions and Bioluminescence Measurements

*Photobacterium kishitanii* ANT‐2200, formerly described as *P. phosphoreum* ANT‐2200, was used as the wild‐type strain. This strain was isolated from Mediterranean deep‐sea waters at 2200 m depth (al Ali et al. 2010; Bjornsdottir‐Butler et al. 2016; Machado and Gram 2017). The spontaneous low‐bioluminescent mutant DimLum was isolated during a controlled fermenter experiment. All strains and plasmids used in this study are listed in Table S5. *Photobacterium* strains were grown in modified ZoBell medium (al Ali et al. 2010) at 19°C with shaking, while *Escherichia coli* strains were grown in LB at 37°C. For cloning and conjugation, *E. coli* JM109, *E. coli* CC118 λpir, and *E. coli* CC118 λpir carrying pEVS104 were used as previously described (Yanisch‐Perron et al. 1985; Herrero et al. 1990; Stabb and Ruby 2002). When required, antibiotics were added at standard concentrations. Solid media contained 15 g L^−1^ agar, and strains were stored at −80°C in 30% glycerol.

For complementation assays, DimLum was transformed with either pVSV104‐*tfo*X, designated pCLCL‐*tfo*X1, or the empty pVSV104 vector. Transformants were selected on modified ZoBell agar supplemented with kanamycin. Growth was monitored by measuring optical density at 600 nm (OD_600_) using a VWR PV4 UV/Visible spectrophotometer (VWR International). Bioluminescence was measured using an H7155 photomultiplier tube coupled to a C8855 photon counting unit (Hamamatsu), with a 10 ms gate time and expressed as detected photon counts s^−1^.

### 3.2 Plasmid Construction and Genetic Complementation

The wild‐type *tfo*X gene, including 294 bp upstream of the start codon, was amplified from *P. kishitanii* ANT‐2200 genomic DNA using primers listed in Table S6. The PCR product was cloned into pGEM‐T Easy, generating pCLCL‐*tfo*X, and verified by Sanger sequencing. The NcoI–SacI fragment was then subcloned into the broad‐host‐range vector pVSV104, which carries a kanamycin resistance marker (Dunn et al. 2006), to generate pCLCL‐*tfo*X1.

Plasmid transfer into DimLum was performed by triparental conjugation using *E. coli* CC118 λpir carrying pCLCL‐*tfo*X1 as donor and *E. coli* CC118 λpir carrying pEVS104 as helper strain, as described by Stabb and Ruby (2002). Donor, helper, and recipient cultures were mixed at a 1:1:1 ratio, spotted onto nonselective ZoBell agar, incubated at 28°C, and then plated on selective ZoBell agar containing kanamycin. Empty‐vector controls were generated in parallel.

### 3.3 Whole‐Genome Sequencing and Variant Analysis

Genomic DNA from WT and DimLum strains was sequenced by MGX Genomics using Illumina MiniSeq paired‐end sequencing. Reads were mapped to the *P. kishitanii* ANT‐2200 reference genome, GenBank accession GCA_000617925.1, using BWA‐MEM. Low‐quality alignments were removed with SAMtools; variants were called with Octopus, and annotations were performed with SnpEff. Only high‐confidence variants with QUAL > 30 and DP > 10 were retained for analysis.

### 3.4 RNA Extraction, RNA‐Seq, and RT‐qPCR

Total RNA was extracted from independent replicate cultures harvested at defined growth stages, as described by Simon et al. (2025). RNA quality was assessed by NanoDrop and TapeStation, and the absence of genomic DNA was confirmed using no‐RT controls. Growth stages used for RNA‐seq, and RT‐qPCR are detailed in Tables S7 and S8.

For RNA‐seq, stranded libraries were prepared and sequenced by MGX Genomics on an Illumina MiniSeq platform. Reads were trimmed with fastp, aligned to the reference genome with STAR, and assigned to annotated features using featureCounts. Differential expression analysis was performed with DESeq2 using an adjusted *p* value < 0.05 and an absolute log_2_FC ≥ 2.

For RT‐qPCR validation, total RNA was reverse‐transcribed using PrimeScript RT (Takara), and quantitative PCR was performed using SYBR Green chemistry (SsoAdvanced Universal SYBR Green Supermix; Bio‐Rad). Gene expression levels were normalized to *rpo*D and calculated using the ΔΔ*C*_t_ method (Livak and Schmittgen 2001). Each sample was analyzed in three technical replicates, with no‐template controls included. Primer sequences are listed in Table S6.

## Author Contributions

**Marc Garel:** supervision, conceptualization, methodology, investigation, validation. **Gwenola Simon:** conceptualization, methodology, supervision, validation, funding acquisition, investigation, project administration. **Laurie Casalot:** conceptualization, methodology, validation, investigation, funding acquisition, project administration, supervision. **Elisa Lefeuvre:** methodology, investigation. **Capucine Le Cam Ligier:** conceptualization, methodology, validation, investigation, funding acquisition, project administration, supervision. **Charlotte Berthelier:** formal analysis, methodology, visualization. **Corinne Valette:** methodology, conceptualization.

## Funding

The project leading to this publication has received funding from the European FEDER Fund under project 1166‐39417.

## Ethics Statement

This article does not contain any studies with human participants performed by any of the authors.

## Conflicts of Interest

The authors declare no conflicts of interest.

## Supporting information


**Figure S1:** RT‐qPCR analysis of selected genes from the lux–rib operon across different growth points. Expression levels are shown as log_2_ fold changes relative to the WT reference strain (set to 0), after normalization to the housekeeping gene rpoD. Measurements were performed at three growth points (T1, T2, and T3). Bars represent mean values across biological replicates, with error bars indicating the standard error of the mean (SEM). The tfoX‐complemented strain, DimLum (pCLCL tfoX1)) is shown in blue, and the DimLum mutant is shown in pink.

**Table S1:** Growth and bioluminescence‐parameters in *Photobacterium kishitanii* ANT‐2200 were calculated for each strain from kinetic measurements. DOmax corresponds to the maximum optical density at 600 nm (OD_600_), reflecting the maximal biomass reached. BLmax corresponds to the maximum bioluminescence intensity, expressed as photons·s^−1^. qBLmax represents the maximal specific bioluminescence production rate, calculated as the maximal rate of light production normalized to biomass. Qx corresponds to the specific growth rate, estimated from the slope of the linear regression of ln(OD600) versus time during the exponential growth phase. Values are presented as mean ± SEM from independent biological replicates, with the number of biological replicates indicated as “n_bio.” Statistical significance was assessed using Welch's *t*‐test comparing each strain to WT for the same parameter, followed by Benjamini–Hochberg correction across all statistical tests reported in the table. Adjusted *p* values are shown as “*p*_adj_global”. Significance categories are indicated as follows: ns, not significant; *, adjusted *p* < 0.05; **, adjusted *p* < 0.01; ***, adjusted *p* < 0.001.

**Table S2:** Gene expression levels were compared between DimLum and wild‐type (WT) samples for each annotated feature. Geneid corresponds to the unique gene identifier, and replicon indicates the genomic replicon. feature_type specifies the annotated feature class. The columns gene and product provide gene names and functional annotations when available, while label corresponds to the annotation used for visualization. WT and DimLum represent raw read counts for each condition at the corresponding time point. meanCount corresponds to the average read count across both conditions, and time specifies the sampling time point (T1–T3). log_2_FC indicates the log_2_‐transformed fold change in expression (DimLum vs. WT), with positive values indicating higher expression in DimLum and negative values indicating reduced expression. *y* represents the negative log_10_‐transformed *p* value (−log10(*p*value)), reflecting the statistical significance of differential expression. Category indicates the direction of regulation based on a predefined log_2_ fold‐change threshold.

**Table S3:** Variants identified from whole‐genome sequencing in the DimLum strain relative to the wild‐type (WT) reference are reported with their genomic position and predicted functional effects. Chromosome indicates the genomic replicon, and position corresponds to the nucleotide coordinate of the variant. Variant specifies the type of mutation. Reference and alternative denote the reference and mutated nucleotides, respectively. Effect describes the predicted functional consequence of the variant based on genomic annotation, and impact indicates the predicted severity. Gene corresponds to the affected gene or genomic feature, and prot_change indicates the predicted amino acid change when applicable. Function indicates the predicted or annotated function of the affected gene.

**Table S4:** RT‐qPCR analysis of gene expression in *Photobacterium kishitanii* ANT‐2200. Expression levels are reported for each target gene, strain, and growth point. The strains analyzed include WT, DimLum, DimLum(pCLCL‐tfoX1), and T‐, and growth points T1, T2, and T3 correspond to the three sampled stages along the growth curve. Relative expression was calculated using the Δ*C*_t_/ΔΔ*C*_t_ method with *rpoD* as the housekeeping gene and WT as the reference strain at the corresponding growth point. Values are expressed as log_2_ fold change relative to WT and shown as mean ± SEM from independent biological replicates (“n_bio”), with individual replicate values provided in separate columns. Statistical significance was assessed on Δ*C*_t_ values using Welch's *t*‐test comparing each strain to WT for the same target gene and growth point. Raw *p* values and Benjamini–Hochberg adjusted *p* values are reported. “*p*_adj_by_target” indicates correction within each target gene, whereas “*p*_adj_global” indicates correction across all tests in the table. Significance categories are: ns, not significant; *, *p* < 0.05; **, *p* < 0.01; ***, *p* < 0.001.

**Table S5:** Bacterial strains and plasmids used in this study. The table lists the *Photobacterium kishitanii* ANT‐2200 wild‐type strain and derivatives, the *Escherichia coli* strains used for cloning and conjugation, and the plasmids used or constructed in this work. Relevant characteristics and references are indicated for each strain or plasmid.

**Table S6:** Primers used in this study; F, Forward; R, Reverse.

**Table S7:** RNA sequencing samples used in this study are described according to strain, growth phase, and biological replication. Sample ID corresponds to the unique identifier assigned to each RNA sample. Strain indicates the bacterial strain analyzed. Growth phase describes the physiological state of the culture at the time of sampling, and timepoint label (T1–T3) refers to the corresponding position along the growth curve. Concentration (ng/μL) indicates the RNA concentration measured prior to library preparation.

**Table S8:** RT‐qPCR samples used in this study are described according to strain, growth conditions, and biological replication. Sample ID corresponds to the unique identifier assigned to each sample. Strain indicates the bacterial strain analyzed. Growth phase describes the physiological state of the culture at the time of sampling, and timepoint label (T1–T3) refers to defined positions along the growth curve. OD_600_ indicates the optical density at 600 nm measured at the time of sampling, reflecting cell density, and time (min) corresponds to the sampling time during the growth experiment. Biological replicate refers to independent cultures processed under identical conditions.

## Data Availability

Raw genome sequencing reads for Lum and DimLum were deposited in the NCBI Sequence Read Archive (SRA) under BioProject accession PRJNA1464527. RNA‐seq raw reads and processed count matrices were deposited in GEO (Gene Expression Omnibus) under accession GSE330586. Custom scripts used for comparative genomics, RNA‐seq analyses, and figure generation are available on GitHub at https://github.com/clecamligier/tfoX_bioluminescence_Pkishitanii. Raw qPCR data and plasmid constructs are available upon request.

## References

[mmi70104-bib-0001] al Ali, B. , M. Garel , P. Cuny , et al. 2010. “Luminous Bacteria in the Deep‐Sea Waters Near the ANTARES Underwater Neutrino Telescope (Mediterraean Sea).” Chemical Ecology 26: 57–72. 10.1080/02757540903513766.

[mmi70104-bib-0002] Ast, J. C. , I. Cleenwerck , K. Engelbeen , et al. 2007. “ *Photobacterium kishitanii* sp. Nov., a Luminous Marine Bacterium Symbiotic With Deep‐Sea Fishes.” International Journal of Systematic and Evolutionary Microbiology 57: 2073–2078. 10.1099/ijs.0.65153-0. 17766874

[mmi70104-bib-0003] Baldwin, T. O. , J. H. Devine , R. C. Heckel , J.‐W. Lin , and G. S. Shadel . 1989. “The Complete Nucleotide Sequence of the *lux* Regulon of *Vibrio fischeri* and the *luxABN* Region of *Photobacterium leiognathi* and the Mechanism of Control of Bacterial Bioluminescence.” Journal of Bioluminescence and Chemiluminescence 4: 326–341. 10.1002/bio.1170040145. 2801220

[mmi70104-bib-0004] Ball, A. S. , R. R. Chaparian , and J. C. van Kessel . 2017. “Quorum Sensing Gene Regulation by LuxR/HapR Master Regulators in Vibrios.” Journal of Bacteriology 199: e00105‐17. 10.1128/jb.00105-17. PMC5585708 28484045

[mmi70104-bib-0005] Bellissimo, K. A. , A. N. Septer , C. A. Whistler , C. Rodríguez , and E. V. Stabb . 2024. “Deletion of luxI Increases Luminescence of *Vibrio fischeri* .” MBio 15: e02446‐24. 10.1128/mbio.02446-24. PMC11481858 39315803

[mmi70104-bib-0006] Bjornsdottir‐Butler, K. , S. A. McCarthy , P. V. Dunlap , and R. A. Benner . 2016. “ *Photobacterium angustum* and *Photobacterium kishitanii* , Psychrotrophic High‐Level Histamine‐Producing Bacteria Indigenous to Tuna.” Applied and Environmental Microbiology 82: 2167–2176. 10.1128/AEM.02833-15. PMC4807525 26826233

[mmi70104-bib-0007] Bloom‐Ackermann, Z. , S. Navon , H. Gingold , R. Towers , Y. Pilpel , and O. Dahan . 2014. “A Comprehensive tRNA Deletion Library Unravels the Genetic Architecture of the tRNA Pool.” PLoS Genetics 10: e1004084. 10.1371/journal.pgen.1004084. PMC3894157 24453985

[mmi70104-bib-0008] Brodl, E. , A. Csamay , C. Horn , J. Niederhauser , H. Weber , and P. Macheroux . 2020. “The Impact of LuxF on Light Intensity in Bacterial Bioluminescence.” Journal of Photochemistry and Photobiology. B 207: 111881. 10.1016/j.jphotobiol.2020.111881. 32325406

[mmi70104-bib-0009] Brodl, E. , A. Winkler , and P. Macheroux . 2018. “Molecular Mechanisms of Bacterial Bioluminescence.” Computational and Structural Biotechnology Journal 16: 551–564. 10.1016/j.csbj.2018.11.003. PMC6279958 30546856

[mmi70104-bib-0010] Cameron, A. D. S. , and R. J. Redfield . 2006. “Non‐Canonical CRP Sites Control Competence Regulons in Escherichia Coli and Many Other γ‐Proteobacteria.” Nucleic Acids Research 34: 6001–6014. 10.1093/nar/gkl734. PMC1635313 17068078

[mmi70104-bib-0011] Dalia, A. B. , D. W. Lazinski , and A. Camilli . 2014. “Identification of a Membrane‐Bound Transcriptional Regulator That Links Chitin and Natural Competence in *Vibrio cholerae* .” MBio 5: e01028‐13. 10.1128/mbio.01028-13. PMC3903286 24473132

[mmi70104-bib-0012] Dunlap, P. 2014. “Biochemistry and Genetics of Bacterial Bioluminescence.” Advances in Biochemical Engineering/Biotechnology 144: 37–64. 10.1007/978-3-662-43385-0_2. 25084994

[mmi70104-bib-0013] Dunn, A. K. , D. S. Millikan , D. M. Adin , J. L. Bose , and E. V. Stabb . 2006. “New Rfp‐ and pES213‐Derived Tools for Analyzing Symbiotic *Vibrio fischeri* Reveal Patterns of Infection and Lux Expression In Situ.” Applied and Environmental Microbiology 72: 802–810. 10.1128/AEM.72.1.802-810.2006. PMC1352280 16391121

[mmi70104-bib-0014] Dunn, A. K. , B. A. Rader , E. V. Stabb , and M. J. Mandel . 2015. “Regulation of Bioluminescence in *Photobacterium leiognathi* Strain KNH6.” Journal of Bacteriology 197: 3676–3685. 10.1128/jb.00524-15. PMC4626902 26350139

[mmi70104-bib-0015] Eckstein, S. , J. Brehm , M. Seidel , M. Lechtenfeld , and R. Heermann . 2021. “Two Novel XRE‐Like Transcriptional Regulators Control Phenotypic Heterogeneity in *Photorhabdus luminescens* Cell Populations.” BMC Microbiology 21: 63. 10.1186/s12866-021-02116-2. PMC7905540 33627070

[mmi70104-bib-0016] Fuertes‐Perez, S. , R. F. Vogel , and M. Hilgarth . 2021. “Comparative Genomics of Photobacterium Species From Terrestrial and Marine Habitats.” Current Research in Microbial Sciences 2: 100087. 10.1016/j.crmicr.2021.100087. PMC8671102 34950912

[mmi70104-bib-0017] Gould, A. L. , S. A. Donohoo , E. D. Román , and E. E. Neff . 2023. “Strain‐Level Diversity of Symbiont Communities Between Individuals and Populations of a Bioluminescent Fish.” ISME Journal 17: 2362–2369. 10.1038/s41396-023-01550-6. PMC10689835 37891426

[mmi70104-bib-0018] Haddock, S. H. D. , M. A. Moline , and J. F. Case . 2010. “Bioluminescence in the Sea.” Annual Review of Marine Science 2: 443–493. 10.1146/annurev-marine-120308-081028. 21141672

[mmi70104-bib-0019] Herrero, M. , V. de Lorenzo , and K. N. Timmis . 1990. “Transposon Vectors Containing Non‐Antibiotic Resistance Selection Markers for Cloning and Stable Chromosomal Insertion of Foreign Genes in Gram‐Negative Bacteria.” Journal of Bacteriology 172: 6557–6567. 10.1128/jb.172.11.6557-6567.1990. PMC526845 2172216

[mmi70104-bib-0020] Katznelson, R. , and S. Ulitzur . 1977. “Control of Luciferase Synthesis in a Newly Isolated Strain of *Photobacterium leiognathi* .” Archives of Microbiology 115: 347–351. 10.1007/BF00446462. 603341

[mmi70104-bib-0021] Konovalova, A. , L. Søgaard‐Andersen , and L. Kroos . 2014. “Regulated Proteolysis in Bacterial Development.” FEMS Microbiology Reviews 38: 493–522. 10.1111/1574-6976.12050. PMC4002673 24354618

[mmi70104-bib-0022] Livak, K. J. , and T. D. Schmittgen . 2001. “Analysis of Relative Gene Expression Data Using Real‐Time Quantitative PCR and the 2−ΔΔCT Method.” Methods 25: 402–408. 10.1006/meth.2001.1262. 11846609

[mmi70104-bib-0023] Lo Scrudato, M. , and M. Blokesch . 2013. “A Transcriptional Regulator Linking Quorum Sensing and Chitin Induction to Render *Vibrio cholerae* Naturally Transformable.” Nucleic Acids Research 41: 3644–3658. 10.1093/nar/gkt041. PMC3616704 23382174

[mmi70104-bib-0024] Machado, H. , and L. Gram . 2017. “Comparative Genomics Reveals High Genomic Diversity in the Genus *Photobacterium* .” Frontiers in Microbiology 8: 1204. 10.3389/fmicb.2017.01204. PMC5489566 28706512

[mmi70104-bib-0025] Mahmoud, S. A. , and P. Chien . 2018. “Regulated Proteolysis in Bacteria.” Annual Review of Biochemistry 87: 677–696. 10.1146/annurev-biochem-062917-012848. PMC6013389 29648875

[mmi70104-bib-0026] Meighen, E. A. 1993. “Bacterial Bioluminescence: Organization, Regulation, and Application of the Lux Genes.” FASEB Journal: Official Publication of the Federation of American Societies for Experimental Biology 7: 1016–1022. 10.1096/fasebj.7.11.8370470. 8370470

[mmi70104-bib-0027] Metzger, L. C. , N. Matthey , C. Stoudmann , E. J. Collas , and M. Blokesch . 2019. “Ecological Implications of Gene Regulation by TfoX and TfoY Among Diverse *Vibrio* Species.” Environmental Microbiology 21: 2231–2247. 10.1111/1462-2920.14562. PMC6618264 30761714

[mmi70104-bib-0028] Metzger, L. C. , S. Stutzmann , T. Scrignari , C. V. der Henst , N. Matthey , and M. Blokesch . 2016. “Independent Regulation of Type VI Secretion in *Vibrio cholerae* by TfoX and TfoY.” Cell Reports 15: 951–958. 10.1016/j.celrep.2016.03.092. PMC4858559 27117415

[mmi70104-bib-0029] Moi, I. M. , N. N. Roslan , A. T. C. Leow , et al. 2017. “The Biology and the Importance of *Photobacterium* Species.” Applied Microbiology and Biotechnology 101: 4371–4385. 10.1007/s00253-017-8300-y. 28497204

[mmi70104-bib-0030] O'Grady, E. A. , and C. F. Wimpee . 2008. “Mutations in the Lux Operon of Natural Dark Mutants in the Genus *Vibrio* .” Applied and Environmental Microbiology 74: 61–66. 10.1128/AEM.01199-07. PMC2223213 17981944

[mmi70104-bib-0031] Qin, N. , S. M. Callahan , P. V. Dunlap , and A. M. Stevens . 2007. “Analysis of LuxR Regulon Gene Expression During Quorum Sensing in *Vibrio fischeri* .” Journal of Bacteriology 189: 4127–4134. 10.1128/jb.01779-06. PMC1913387 17400743

[mmi70104-bib-0032] Ramesh, C. H. , and R. Mohanraju . 2017. “Genetic Diversity of Bioluminescent Bacteria in Diverse Marine Niches.” Indian Journal of Geo‐Marine Sciences 46: 2054–2062.

[mmi70104-bib-0033] Schubert, R. A. , I. B. Dodd , J. B. Egan , and K. E. Shearwin . 2007. “Cro's Role in the CI–Cro Bistable Switch Is Critical for Λ's Transition From Lysogeny to Lytic Development.” Genes & Development 21: 2461–2472. 10.1101/gad.1584907. PMC1993876 17908932

[mmi70104-bib-0034] Simon, G. , L. Casalot , C. Valette , C. Burot , J. F. Rontani , and P. Bonin . 2025. “Do Carotenoids Protect Phytodetritus‐Associated Bacteria From Oxidative Stress?” Environmental Science and Pollution Research 32: 11167–11178. 10.1007/s11356-025-36080-5. PMC12014824 40198437

[mmi70104-bib-0035] Søndberg, E. , A. K. Sinha , K. Gerdes , and S. Semsey . 2019. “CRP Interacts Specifically With Sxy to Activate Transcription in *Escherichia coli* .” Frontiers in Microbiology 10: 2053. 10.3389/fmicb.2019.02053. PMC6728893 31543875

[mmi70104-bib-0036] Stabb, E. V. , and E. G. Ruby . 2002. “RP4‐Based Plasmids for Conjugation Between Escherichia Coli and Members of the Vibrionaceae.” In Methods in Enzymology, 413–426. Elsevier. 10.1016/S0076-6879(02)58106-4. 12474404

[mmi70104-bib-0037] Stevens, A. M. , and E. P. Greenberg . 1997. “Quorum Sensing in *Vibrio fischeri* : Essential Elements for Activation of the Luminescence Genes.” Journal of Bacteriology 179: 557–562. 10.1128/jb.179.2.557-562.1997. PMC178731 8990313

[mmi70104-bib-0038] Sun, Y. , E. E. Bernardy , B. K. Hammer , and T. Miyashiro . 2013. “Competence and Natural Transformation in Vibrios.” Molecular Microbiology 89: 583–595. 10.1111/mmi.12307. PMC3820095 23803158

[mmi70104-bib-0039] Sung, N.‐D. , and C. Y. Lee . 2004. “Coregulation of Lux Genes and Riboflavin Genes in Bioluminescent Bacteria of *Photobacterium phosphoreum* .” Journal of Microbiology, Seoul, Korea 42: 194–199. 15459647

[mmi70104-bib-0040] Tanet, L. , S. Martini , L. Casalot , and C. Tamburini . 2020. “Reviews and Syntheses: Bacterial Bioluminescence—Ecology and Impact in the Biological Carbon Pump.” Biogeosciences 17: 3757–3778. 10.5194/bg-17-3757-2020.

[mmi70104-bib-0041] Tanet, L. , C. Tamburini , C. Baumas , M. Garel , G. Simon , and L. Casalot . 2019. “Bacterial Bioluminescence: Light Emission in *Photobacterium phosphoreum* Is Not Under Quorum‐Sensing Control.” Frontiers in Microbiology 10: 365. 10.3389/fmicb.2019.00365. PMC6409340 30886606

[mmi70104-bib-0042] Urbanczyk, H. , J. C. Ast , and P. V. Dunlap . 2011. “Phylogeny, Genomics, and Symbiosis of Photobacterium.” FEMS Microbiology Reviews 35: 324–342. 10.1111/j.1574-6976.2010.00250.x. 20883503

[mmi70104-bib-0043] Urbanczyk, H. , J. C. Ast , A. J. Kaeding , J. D. Oliver , and P. V. Dunlap . 2008. “Phylogenetic Analysis of the Incidence of Lux Gene Horizontal Transfer in Vibrionaceae.” Journal of Bacteriology 190: 3494–3504. 10.1128/JB.00101-08. PMC2394989 18359809

[mmi70104-bib-0044] Verma, S. C. , and T. Miyashiro . 2013. “Quorum Sensing in the Squid‐Vibrio Symbiosis.” International Journal of Molecular Sciences 14: 16386–16401. 10.3390/ijms140816386. PMC3759917 23965960

[mmi70104-bib-0045] Widder, E. A. 2010. “Bioluminescence in the Ocean: Origins of Biological, Chemical, and Ecological Diversity.” Science 328: 704–708. 10.1126/science.1174269. 20448176

[mmi70104-bib-0046] Wu, R. , M. Zhao , J. Li , H. Gao , B. Kan , and W. Liang . 2015. “Direct Regulation of the Natural Competence Regulator Gene tfoX by Cyclic AMP (cAMP) and cAMP Receptor Protein (CRP) in Vibrios.” Scientific Reports 5: 14921. 10.1038/srep14921. PMC4595672 26442598

[mmi70104-bib-0047] Yamamoto, S. , H. Izumiya , J. Mitobe , et al. 2011. “Identification of a Chitin‐Induced Small RNA That Regulates Translation of the tfoX Gene, Encoding a Positive Regulator of Natural Competence in *Vibrio cholerae* .” Journal of Bacteriology 193: 1953–1965. 10.1128/JB.01340-10. PMC3133033 21317321

[mmi70104-bib-0048] Yanisch‐Perron, C. , J. Vieira , and J. Messing . 1985. “Improved M13 Phage Cloning Vectors and Host Strains: Nucleotide Sequences of the M13mp18 and pUC19 Vectors.” Gene 33: 103–119. 10.1016/0378-1119(85)90120-9. 2985470

