# Supplementary material for: A Spontaneous Mutation in tfoX Impairs Bioluminescence in Photobacterium kishitanii ANT‐2200
Source: Mol Microbiol. 2026 Jul 25;126(4):343–50. doi: 10.1111/mmi.70104 (PMC13644369; doi:10.1111/mmi.70104)
Supplement: Supplementary file 1 [file MMI-126-343-s003.pdf]

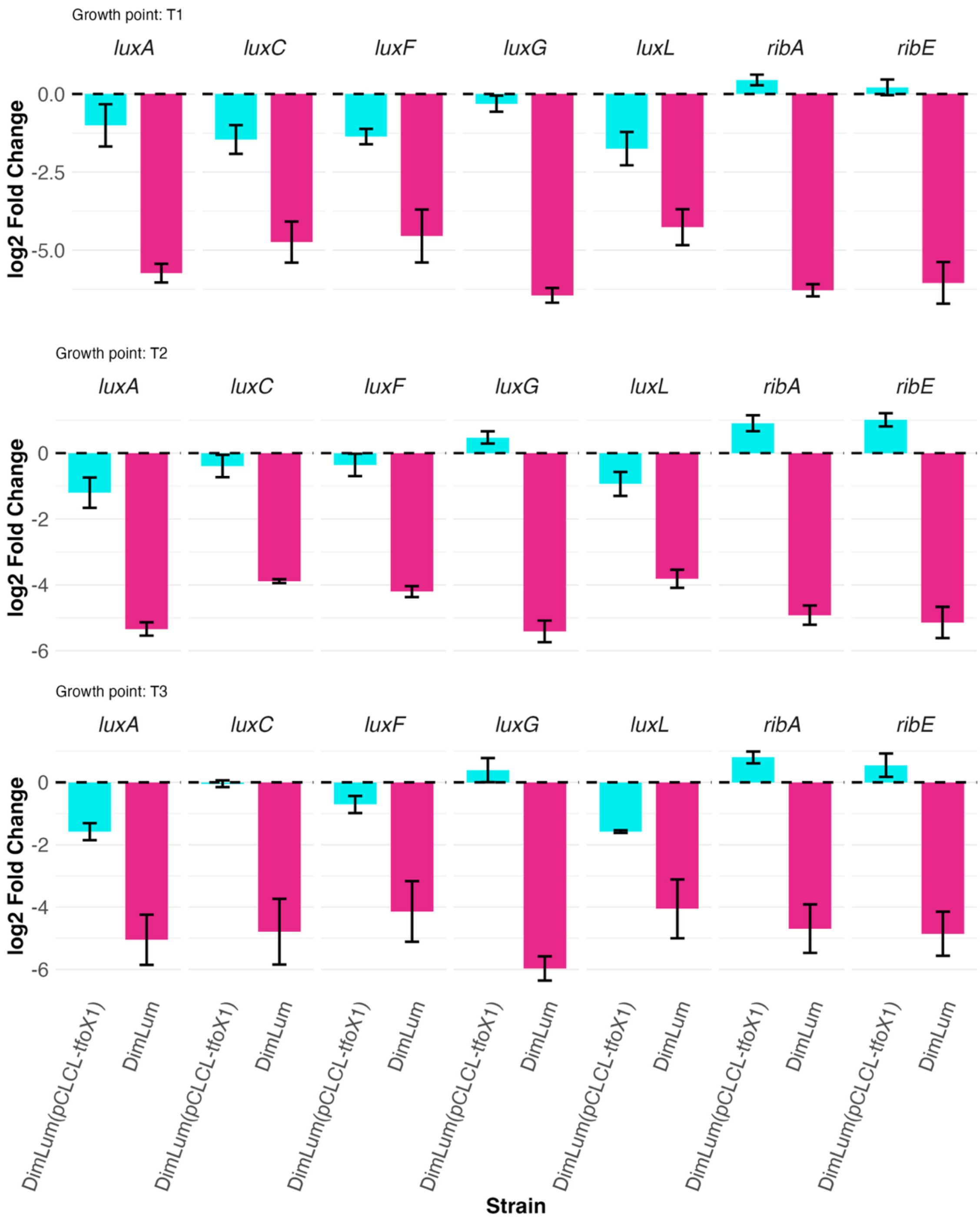

Supplementary Figure S1: RT-qPCR analysis of selected genes from the *lux–rib* operon across different growth points. Expression levels are shown as $\log_2$ fold changes relative to the WT reference strain (set to 0), after normalization to the housekeeping gene *rpo*D. Measurements were performed at three growth points (T1, T2, and T3). Bars represent mean values across biological replicates, with error bars indicating the standard error of the mean (SEM). The tfoX-complemented strain, DimLum (pCLCL-*tfo*X1)) is shown in blue, and the DimLum mutant is shown in pink.
